# Ensemble modeling to predict habitat suitability for a large-scale disturbance specialist

**DOI:** 10.1002/ece3.790

**Published:** 2013-10-06

**Authors:** Quresh S Latif, Victoria A Saab, Jonathan G Dudley, Jeff P Hollenbeck

**Affiliations:** 1 Rocky Mountain Research Station, U.S. Forest Service1648 S. 7th Ave., Bozeman, Montana, 59717; 2 Rocky Mountain Research Station, U.S. Forest Service322 E. Front St., Suite 401, Boise, Idaho, 83702; 3 USGS Forest and Rangeland Ecosystem Science Center3200 SW Jefferson Way, Corvallis, Oregon, 97331

**Keywords:** Black-backed Woodpeckers, forested habitat management, habitat suitability models, Mahalanobis *D*^2^ models, Maxent models, model prediction in no-analogue environments, *Picoides arcticus*, resource selection models, species distribution models, wildfire

## Abstract

To conserve habitat for disturbance specialist species, ecologists must identify where individuals will likely settle in newly disturbed areas. Habitat suitability models can predict which sites at new disturbances will most likely attract specialists. Without validation data from newly disturbed areas, however, the best approach for maximizing predictive accuracy can be unclear (Northwestern U.S.A.). We predicted habitat suitability for nesting Black-backed Woodpeckers (*Picoides arcticus*; a burned-forest specialist) at 20 recently (≤6 years postwildfire) burned locations in Montana using models calibrated with data from three locations in Washington, Oregon, and Idaho. We developed 8 models using three techniques (weighted logistic regression, Maxent, and Mahalanobis *D*^2^ models) and various combinations of four environmental variables describing burn severity, the north–south orientation of topographic slope, and prefire canopy cover. After translating model predictions into binary classifications (0 = low suitability to unsuitable, 1 = high to moderate suitability), we compiled “ensemble predictions,” consisting of the number of models (0–8) predicting any given site as highly suitable. The suitability status for 40% of the area burned by eastside Montana wildfires was consistent across models and therefore robust to uncertainty in the relative accuracy of particular models and in alternative ecological hypotheses they described. Ensemble predictions exhibited two desirable properties: (1) a positive relationship with apparent rates of nest occurrence at calibration locations and (2) declining model agreement outside surveyed environments consistent with our reduced confidence in novel (i.e., “no-analogue”) environments. Areas of disagreement among models suggested where future surveys could help validate and refine models for an improved understanding of Black-backed Woodpecker nesting habitat relationships. Ensemble predictions presented here can help guide managers attempting to balance salvage logging with habitat conservation in burned-forest landscapes where black-backed woodpecker nest location data are not immediately available. Ensemble modeling represents a promising tool for guiding conservation of large-scale disturbance specialists.

## Introduction

Ecologists use habitat suitability models to inform management activities aimed at species conservation (Barrows et al. 2008; Keenan et al. 2011; reviewed by Elith and Leathwick 2009). Most models use site-use data to quantify the environmental distribution of a species, based upon which sites (usually pixels) are assigned ranks according to their relative suitability (hereafter, habitat suitability indices; HSIs). To the extent that a model reflects environmental requisites shaping species' occurrence, HSIs will estimate the species' geographic distribution (Hirzel et al. 2006; Barrows et al. 2008). Habitat models are often used to predict distributions outside sampled areas (Wilson et al. 2005; Elith et al. 2011; except see Zurell et al. 2012), but are typically less accurate when used for this purpose (Heikkinen et al. 2012). Nevertheless, model-based predictions may provide the best available information for guiding management activities in areas where data are not immediately available (the alternative being expert knowledge). By ignoring potentially important complexities of ecological systems, such as biotic interactions or behavioral dynamics, habitat suitability models are typically limited in the information they provide (Zurell et al. 2009; Aarts et al. 2013). Nevertheless, these models can provide a useful first approximation of habitat relationships underlying a species' distribution (Pearson and Dawson 2003).

Predictive accuracy of habitat suitability models depends largely on the data analyzed, the habitat variables used for modeling, and underlying model structure (Austin 2007). Available data used for model development may sample a restricted environmental range, preventing complete description of ecological relationships shaping habitat use (Thuiller et al. 2004). Additionally, without complete knowledge of processes underlying habitat selection, the environmental variables best used for modeling may be unclear. Finally, models often emphasize linear relationships within a resource selection framework (i.e., comparison of habitat use vs. availability), which may not adequately describe relevant ecological relationships (Austin 2007). To deal with these uncertainties, some researchers combine predictions from multiple models using an “ensemble” approach (Araújo and New 2007). By combining models differing in structure, explanatory variables, and data sources, ensemble predictions allow inferences that are robust to uncertainties associated with any individual model. Ensemble modeling has been used to predict responses to climate change, for which data for validating predictions are not immediately available (Araújo and New 2007).

Ensemble modeling requires development of multiple habitat models, a useful exercise in itself. Models that describe observed data equally well but differ in structure can suggest alternative hypotheses regarding underlying species ecology. For example, models describing linear versus nonlinear relationships with a particular habitat feature could fit available data equally well, in which case either could represent the species' true relationship with that feature. Furthermore, differences among models may be most apparent when applied to novel environments (Heikkinen et al. 2012; Wenger and Olden 2012). Application of ensemble predictions may therefore suggest where future data collection efforts could facilitate evaluation of alternative ecological hypotheses, namely where models disagree. Thus, ensemble predictions can help guide future research.

Prediction of habitat suitability is especially necessary to guide conservation of species that specialize on habitats created by large-scale natural disturbance, such as wildfire. Recently disturbed areas provide suitable habitat for specialists for a limited period following disturbance events (e.g., Saab et al. 2007). If during this period, resource extraction opportunities also arise, managers must quickly identify suitable habitat to balance the needs of wildlife and humans. Depending upon phenology and how a species locates new disturbances, colonization of new disturbances may be delayed, impeding timely collection of data for informing management decisions. Data from previous disturbances may therefore provide the only objective information for conservation planning, making model-based prediction a natural approach.

**Figure 1:**
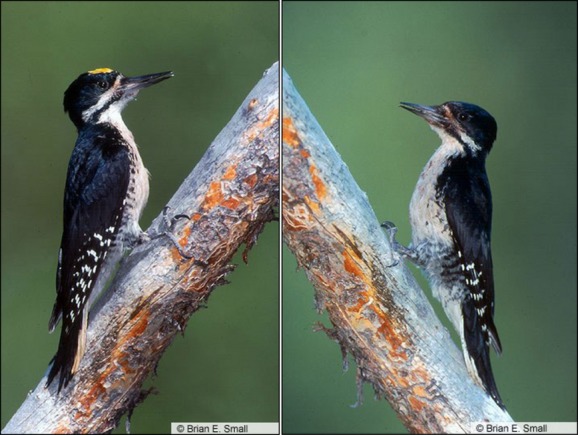
Male (left) and female (right) black-backed woodpeckers (taken from Dixon and Saab 2000).

We developed model-based predictions of habitat suitability for the Black-backed Woodpecker (*Picoides arcticus*; Fig. 1) in Montana east of the Rocky Mountains at 20 recently burned (≤ 6 years postwildfire), dry, mixed-conifer forests. Black-backed woodpeckers specialize on burned-forest habitats (Dixon and Saab 2000). They are almost exclusively restricted to disturbed forests, relatively restricted to burned-forest conditions (Saab et al. 2007, 2011; but see Bonnot et al. 2009), and well-adapted for extracting wood-boring beetle (Cerambycidae and Buprestidae; Beal 1911) larvae from the cambium of burned insect-infested trees (Dixon and Saab 2000). Snags generated by wildfire are often removed by salvage logging to reduce increased risk of recurring wildfire and to provide an economically valuable resource, so forest managers must balance socioeconomic needs with legal requirements to maintain wildlife habitat.

We developed habitat suitability models using three techniques and nest-site data from Washington, Oregon, and Idaho. Models varied in the spatial extent to which they were calibrated, in the environmental variables included, and in the assumed form of relationships with these variables. We used an “ensemble” approach (Araújo and New 2007) to combine predictions from multiple top-performing models that varied in structure and parameterization. We looked for agreement and disagreement among models to consider where habitat suitability inferences were robust to model-specific uncertainties and suggest where future surveys could further our understanding of Black-backed Woodpecker habitat relationships. Given the nature of model-based prediction, we expected predictions to differ among models and agreement to decline when applied to environments differing from the inferential space where models were developed, that is, “no-analogue” environments (Williams and Jackson 2007; Roberts and Hamann 2012; Veloz et al. 2012). We analyzed model predictions to corroborate this expectation and considered its implications for the use of ensemble predictions to guide future research and conservation planning.

## Methods

### Study area

**Figure 2:**
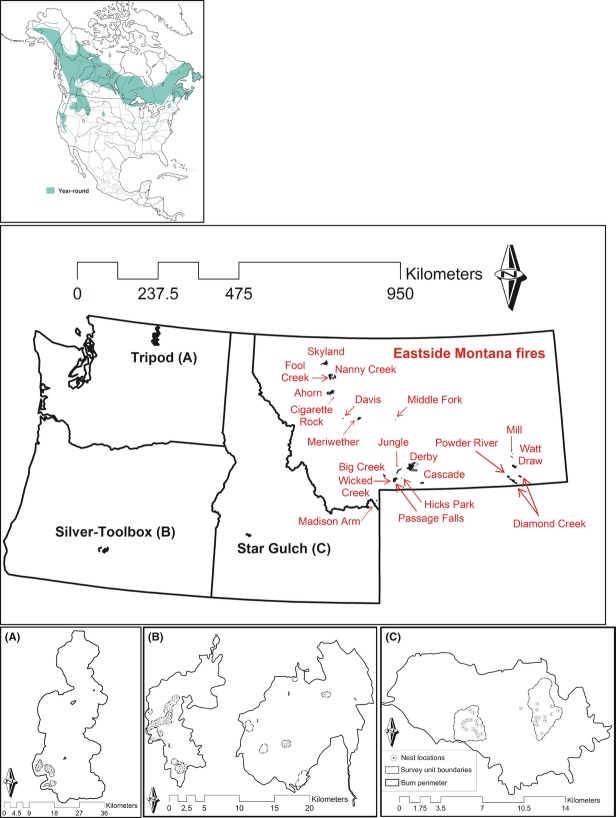
The top map depicts the range for black-backed woodpeckers (taken from Dixon and Saab 2000). The subsequent map illustrates fire locations within state boundaries (Washington, Oregon, Idaho, and Montana; U.S.A.). Bottom maps show nest locations, survey unit boundaries, and burn perimeters at Tripod (A), Silver-Toolbox (B; Silver is west, Toolbox is east), and Star Gulch (C) wildfire locations.

We surveyed wildfires in three western states (U.S.A.) for Black-backed Woodpecker nests: the Tripod Fire in Washington, the Silver and Toolbox fires in Oregon, and the Star Gulch Fire in Idaho (Table 1, Fig. 2). These fires burned in dry, mixed-conifer forest dominated by ponderosa pine (*Pinus ponderosa*) (cf. Schoennagel et al. 2004). Because of the proximity between the Silver and Toolbox fires (ca. 3000 m apart) and concurrent timing (Table 1), we treated them as one location (hereafter the Silver-Toolbox fires). We informed models with nest-site data from these fires and applied the models to predict habitat suitability in similar dry conifer forests at 20 wildfire locations in Montana national forests east of the North American continental divide (2006–2011; Appendix S1).

**Table 1:** Wildfires where Black-backed Woodpecker nests were found and used to develop habitat suitability models

National Forest	Fire Name	Ignition Year	Years surveyed	Full Extent (ha)	Survey Unit Extent (ha)	No. pixels with nests
Okanogan-Wenatchee, Washington	Tripod	2006	2008–2009	99,349	2912	28
Fremont-Winema, Oregon	Silver and Tool Box	2002	2003–2004	33,427	802	44
Boise, Idaho	Star Gulch	1994	1995–1998	12,358	1520	36

### Field surveys for nests

We conducted rectangular belt-transect surveys (averaged 0.2 km × 1 km) within a priori established survey units to identify occupied nest cavities (Dudley and Saab 2003) during early May until late June in multiple years (Table 1), the nesting period for most Black-backed Woodpeckers. Salvage logging impacted increasingly greater portions of the Silver-Toolbox fires over the years (2003–2007). To focus models on prelogging habitat suitability, we excluded data from areas and years impacted by logging (2003–2004 data within 1 km of logged units and all data after 2004 were excluded). We placed the center of belt transects 200 m apart and surveyed 100 m on either side of the center line. Transects began and ended at opposing unit boundaries, so surveys covered each unit. Additionally, nests were opportunistically found just outside originally delineated boundaries, so for this study, we considered surveyed units to include all areas within 250 m of originally established boundaries (Table 1, Fig. 2). We used GPS units (Trimble GeoExplorer 3, Trimble Navigation Limited 1999–2001, Sunnyvale, CA 94085) to determine the geographic coordinates of each nest cavity.

### Environmental variables

We exclusively used remotely sensed variables to maximize applicability to new locations without the need for field measurements. We divided the extent of each fire into 30 × 30-m pixels, and for each pixel, we compiled values for four variables: burn severity (delta normalized burn ratio [ΔNBR]; Key 2006), the north–south orientation of the slope (cosine aspect [COSASP]), and two prefire canopy cover variables compiled at different spatial scales (LocCC and LandCC; Table 2; using ArcGIS 10, ESRI 2010); the latter provided an index of tree or snag density (Russell et al. 2007; Saab et al. 2009; Dudley et al. 2012). The north–south slope orientation relates to a moisture gradient (moist–dry) and thus overall primary productivity. None of the four variables were statistically redundant (LocCC vs. LandCC *r* = 0.635; remaining intercorrelations ≤0.3; Appendix S2). Burn severity and prefire canopy cover variables were averaged across either 1-ha (9 pixels) or 314-ha (1-km radius; 3409 pixels) moving windows centered on each pixel. We considered prefire canopy cover at these two different spatial scales because we suspected snag density could influence suitability of the local area for nesting by affecting nest predation pressure or suitability of a larger area for territory establishment by affecting food resources. Data sources were USGS National Elevation Dataset (1 arc-second; USGS 2012) for cosine aspect and Monitoring Trends in Burn Severity (MTBS 2012) for delta normalized burn ratio. Prefire canopy cover data were originally derived from Landsat Thematic Mapper (TM) images (30 × 30-m resolution), which were further processed by LEMMA (2012; for Tripod and Silver-Toolbox locations), the U.S. Forest Service Remote Sensing Application Center (Johnson et al. 2000; for Star Gulch), or U.S. Forest Service Region 1 Vegetation Mapping Program (VMap v. 11, mid-level data [Berglund et al. 2008]; for eastside Montana locations). Elevation, sine aspect, and slope were also initially considered, but the ecological significance of these variables was less apparent. We therefore restricted our modeling to the above four variables to minimize the potential for over-fitting models.

**Table 2:** Remotely sensed habitat variables used to develop nesting habitat suitability models for Black-backed Woodpeckers

Variable name (abbrev.)	Description
Cosine Aspect (COSASP)	Pixel cosine-transformed orientation of slope (unitless)
Differenced (delta) normalized burn ratio (dNBR)	Median index of burn severity using Landsat TM satellite imagery for 1-ha moving window (unitless)
Percent moderate-to-high canopy cover at local-scale (LocCC)	Proportion of 1-ha moving window with >40% canopy cover
Percent moderate-to-high canopy cover at landscape-scale (LandCC)	Proportion of 314-ha moving window with > 40% canopy cover

### Development and selection of habitat suitability models

Habitat models assumed various relationships with predictor variables: (1) logistic regression models and the simpler of two Maxent models assumed linear relationships (via a model-specific link function), (2) Mahalanobis *D*^2^ models assumed relationships describing optimal suitability at locations matching the average environmental conditions at nest sites, and (3) the more complex Maxent model assumed a mix of linear and nonlinear relationships with key habitat features as supported by data comparing habitat use versus availability. To the extent that models describing different statistical relationships fitted the data reasonably well, they suggested alternative hypotheses regarding ecological relationships determining habitat suitability.

We fitted weighted logistic regression models (hereafter WLR models) to data from each of three locations: Star Gulch, Silver-Toolbox, and Tripod. WLR models analyzed environmental differences between nest and non-nest sites. Non-nest sites were pixels within unit boundaries where nests were never observed during surveys (see Table 1). WLRs employ a weighted distribution to remove the influence of sample size differences between nest and non-nest random sites (Lele and Keim 2006). We used samples of 68 non-nest random sites at the Tripod Fire, 100 at Silver-Toolbox fires, and 47 at the Star Gulch Fire for model development. We constructed models with all possible subsets of the four environmental variables (cf. Russell et al. 2007). For each dataset, we selected the model with the smallest AIC_c_ (Akaike's Information Criterion corrected for small sample size; Burnham and Anderson 2002) to develop ensemble predictions.

Additionally, we constructed Mahalanobis *D*^2^ models (Rotenberry et al. 2006; chronological list of steps provided in Appendix S3). The *D*^2^ statistic described the distance from the multivariate mean of nest sites and was rescaled to provide a habitat suitability index (HSI) with 0–1 range. We derived HSIs from variance partitions derived from principal components analysis applied to nest data (Rotenberry et al. 2006). The *k*th partition is the sum of a sequential subset of *k* principal components. Partition *k*_max_ is the sum of all components and yields a model equivalent to an un-partitioned *D*^2^ model. We derived models from partitions 1–*k*_max_ of all combinations of burn severity, cosine aspect, and prefire canopy cover variables constrained to include burn severity (10 models). We iteratively subsampled the data to equalize representation of the three surveyed wildfire locations in the calibration data. For each of 100 iterations, we sampled 28 nest pixels without replacement from each calibration location (*n* = 84 during each iteration). We used two performance metrics to select models: the median nest HSI (Preston et al. 2008) and AUC (a metric of classification accuracy; Fielding and Bell 1997). A large median nest HSI indicated a restricted environmental distribution of nest sites (Preston et al. 2008), whereas AUC evaluated discrimination of nest pixels from available pixels (represented by 10,000 pixels drawn from within survey unit boundaries, a third from each calibration location). We applied five-fold cross-validation during each iteration, resulting in 500 replicates for each model. We calculated mean performance metrics for the 500 validation datasets (data withheld from each replicate model). When selecting models, we looked for model(s) that achieved both a relatively high median nest HSI and a high AUC. For predicting and mapping habitat suitability, we averaged HSIs across 100 model replicates fitted to subsampled data without cross-validation.

Finally, we developed Maxent (maximum entropy) models (Phillips et al. 2006; Elith et al. 2011). Maxent HSIs describe a probability of species presence (0–1 range, the HSI) conditioned upon the environmental distribution of available pixels and a prevalence of 0.5. To represent available pixels, we drew 10,000 pixels from within study unit boundaries. Following nest-site sample sizes obtained from each location, we drew 2593, 4074, and 3333 pixels from the Tripod, Silver-Toolbox, and Star Gulch locations, respectively (Table 2). We used default regularization settings provided by the Maxent software. Regularization relaxes the need for formal model selection, but exclusion of extraneous or redundant predictor variables is nevertheless recommended (Elith et al. 2011; Merow et al. 2013). We therefore excluded variables that contributed minimally to initial models while monitoring AUC (mean for five validation datasets generated by five-fold cross-validation) to ensure variable exclusion did not cause inordinate losses of explanatory power. When estimating habitat suitability for generating ensemble predictions and maps, we used versions of selected Maxent models fitted to all data without cross-validation.

### Model performance and selection of classification thresholds

We used several performance metrics to assess habitat suitability estimates within surveyed units at the three calibration locations (Tripod, Silver-Toolbox, and Star Gulch). We translated continuous HSI values into a binary classification for each pixel (1 = moderate-to-high suitability habitat [hereafter highly suitable habitat]; 0 = low suitability to unsuitable habitat [hereafter low suitability habitat]) using HSI thresholds that maximized predictive gain across all three calibration locations; predictive gain = sensitivity − (1 − specificity), where sensitivity = the proportion of nest-site pixels classified highly suitable and specificity = the proportion of available pixels classified as low suitability (Browning et al. 2005; Hollenbeck et al. 2011). We then used sensitivity and specificity at selected thresholds and AUCs at each location to evaluate HSIs. We considered AUC > 0.6 to indicate a useful model (Fielding and Bell 1997). AUC is expected to be lower when evaluating discrimination of used versus available sites but can nevertheless be informative (Phillips et al. 2006). We considered reasonable performance indicated by AUCs, sensitivities, and specificities within surveyed units to be necessary for model application outside surveyed areas.

### Ensemble predictions, model agreement, and habitat relationships

We combined predictions into an “ensemble” by calculating the number of models predicting each pixel as highly suitable using selected classification thresholds (see “bounding-box” approach described by Araújo and New 2007). We compared liberal versus conservative ensemble predictions of suitability: the proportion of pixels classified highly suitable by at least one model versus all 8 models at each wildfire location. To quantify model agreement, we examined the proportion of pixels consistently classified highly suitable or low suitability by all models. Where agreement was found, we considered suitability robust to uncertainties in the relative predictive value of particular models. Model predictions that relate positively and linearly with the proportion of pixels used by the species are considered desirable (Jiménez-Valverde et al. 2013). By analogy, we considered such a relationship between ensemble predictions and the proportion of pixels containing nests to be desirable. We used Pearson's correlation coefficient and simple linear regression (cor and lm functions, R v. 2.15.1; R Core Team 2013) to analyze this relationship.

**Figure 3:**
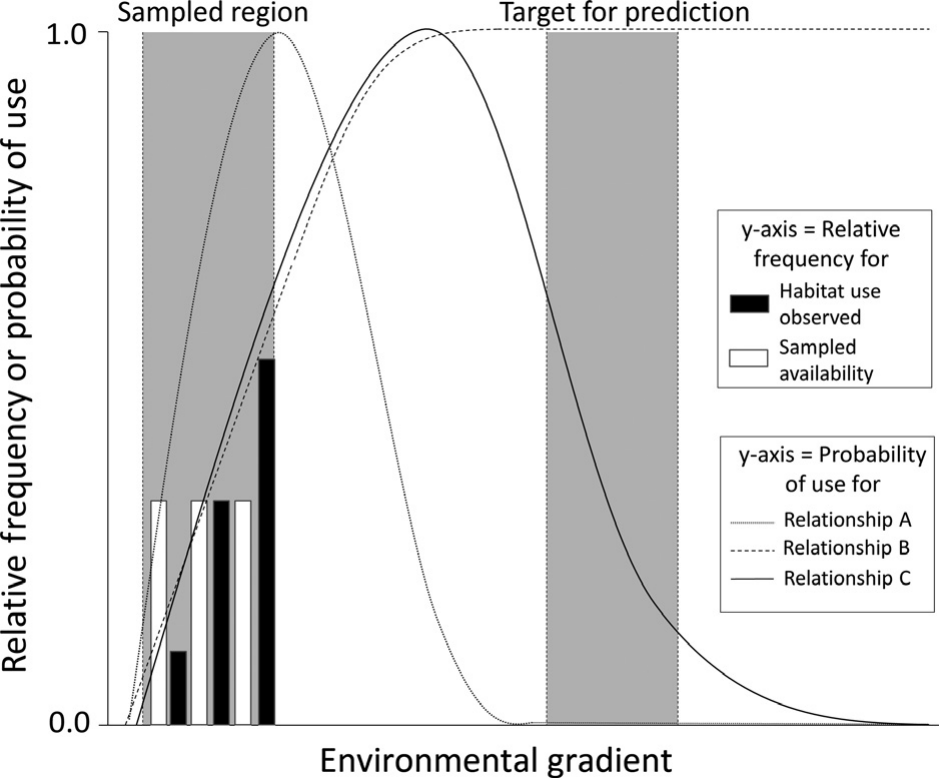
Three hypothetical species–habitat relationships (A, B, C) and use-availability data potentially generated given any of the depicted relationships. Suggested relationships (A, B, C) are purely descriptive and do not assume particular mathematical relationships. The modeling technique best suited for prediction may depend on the form of the true relationship (A, B, or C) governing a species distribution. A distance model reflecting environmental distance from used habitat would be best for predicting a distribution governed by relationship A, whereas a resource selection model, which compares use to availability, would more likely reflect relationship B. Neither model would reflect relationship C. Both types of models would correctly estimate higher suitability at the positive end of this gradient within the sampled range, but model predictions would differ widely outside sampled environments.

For insight into the ecological hypotheses represented by individual models and the behavior of ensemble predictions, we compared model predictions and analyzed patterns in model agreement. We compared habitat relationships represented by different models by plotting mean ± SD HSI values against environmental variables (Hanser 2011). We also compared the proportion of pixels classified highly suitable by each model at individual locations (*n* = 23). We expected predictions to differ among models and model agreement to decline with increasing environmental distance from survey units. Our models represented two distinct types. WLRs and Maxent models represented *resource selection models*, which emphasize differences in used versus available sites (Lele and Keim 2006; Phillips et al. 2006; Elith et al. 2011). Strictly speaking, WLRs analyzed presence–absence data, which are expected to yield maximally informative models (Royle et al. 2012). At the resolution of our data (30 × 30 m), however, “absences” could arise from low prevalence of nests rather than poor habitat quality (in sensu Lobo et al. 2010). We therefore expected data analyzed by WLRs and Maxent models to provide similar information regarding resource selection in this study. In contrast, *distance models* (Mahalanobis *D*^2^) strictly reference environmental dissimilarity from used habitat without reference to availability (Browning et al. 2005; Rotenberry et al. 2006). Distance models will always predict declining suitability with increasing environmental dissimilarity from known species-use locations and, consequently, from sampled environments (Knick and Rotenberry 1998). In contrast, if resource selection favors a particular direction along an environmental gradient within sampled environments, resource selection models will predict increasing suitability outside sampled environments in the favored direction (Fig. 3). Given these tendencies, we expected agreement between resource selection and distance models to decline outside sampled environments, that is, in no-analogue environments. Differences in complexity (number of parameters) can further drive divergence among models because complex models fit calibration data more tightly, potentially reducing predicted suitability by complex models faster outside sampled environments. Resource selection models also differed in underlying structure (Maxent vs. logistic regression) in other ways that can result in somewhat different suitability estimates (Royle et al. 2012). Finally, because calibration locations differed environmentally from each other (Appendix S4), parameter estimates and thus predictions could also differ among models fitted to different datasets.

We related environmental distance from sampled environments (i.e., survey units) with model agreement. We calculated multivariate distances from mean values scaled by the covariance matrix for survey unit pixels (square root of values from “mahalanobis” function in R 2.15.1; R Core Team^2013^). We calculated distances from a sample of 15,000 pixels drawn from within survey unit boundaries (5000 each from Tripod, Silver-Toolbox, and Star Gulch locations) based on all environmental variables (Table 2) to individual wildfire locations (represented by a maximum of 5000 pixels drawn from each location). We used Pearson's correlation and linear regression (cor and lm functions, R v. 2.15.1; R Core Team 2013) to analyze the relationship between location-wide model agreement and median environmental distance from survey units (*n* = 23).

## Results

### Habitat models and their performance at calibration locations

Field surveyors found nests within 108 pixels at the three surveyed locations (Table 1). Top-ranked WLR models for Tripod, Silver-Toolbox, and Star Gulch fires all described relationships with burn severity (ΔNBR; Appendices S5, S6). WLR parameters describing relationships with burn severity were consistently positive. Star Gulch Fire model HSIs also related positively with landscape-scale canopy cover (LandCC), suggesting an affinity for moderate–high postfire snag densities.

We selected three Mahalanobis *D*^2^ models. The model derived from partition 1 of cosine aspect and burn severity variables achieved the highest median nest HSI (0.475), but a low AUC (0.577). The remaining models achieved similar median nest HSIs (0.41–0.44), but three achieved higher AUCs (0.70–0.72): models derived from (1) partition 2 of cosine aspect and burn severity variables, (2) partition 3 of burn severity and prefire canopy cover variables, and (3) partition 4 of all four variables (Appendix S7). All three models arose from the *k*_max_ partition and were therefore equivalent to unpartitioned models. These models reflected average conditions at nest sites (Table 3).

**Table 3:** Descriptive statistics (mean ± SD) for pixels containing Black-backed Woodpecker nests (“used” pixels) and available pixels (drawn randomly from units surveyed for nests)

Variable	Nest (*n* = 84; 28 from each of 3 locations)	Available (*n* = 30,000; 10,000 from each of 3 locations)
COSASP	0.28 ± 0.62	0.00 ± 0.69
ΔNBR	518.1 ± 200.3	327.8 ± 235.2
LocCC	0.82 ± 0.30	0.63 ± 0.42
LandCC	0.65 ± 0.16	0.59 ± 0.19

We retained two Maxent models for ensemble predictions. A maximally parameterized model (allowing linear, quadratic, product, threshold, and hinge relationships with all four variables) achieved a validation AUC = 0.725. Excluding LandCC, which contributed minimally (7%) to this initial model, and allowing only linear, quadratic, and product features did not compromise discriminatory power (AUC = 0.726). Burn severity was the predominant contributor to this 3-variable model (66%; LocCC contributed 18% and COSASP 16%). A model allowing only a linear relationship with burn severity, however, also did not compromise explanatory power (AUC = 0.730). We retained the 3-variable and the ΔNBR-only model for ensemble predictions.

Within survey unit boundaries, all eight selected models were at least moderately informative for discriminating nest from landscape pixels (all site-specific AUCs ≥ 0.65; Table 4). Furthermore, all models classified most nest pixels as highly suitable (all sensitivities ≥ 0.56; all except two ≥ 0.70) while also classifying a substantial portion of the landscape as low suitability (specificities ≥ 0.34; for classification thresholds, see Appendix S8). Most models classified the great majority of nest pixels as highly suitable, so limitations to discriminative power were mainly attributable to limited specificity, which is less of a concern when discriminating used from available habitat (Phillips et al. 2006).

**Table 4:** Evaluation scores of habitat suitability models for nesting Black-backed Woodpeckers. AUC evaluates model ability to discriminate nests from available sites independent of the HSI threshold used for classification. Sensitivity is the proportion of nest pixels correctly classified as highly suitable, and specificity is the proportion of available pixels classified as low suitability habitat at the HSI threshold that maximized predictive gain (sensitivity − [1 − specificity]; thresholds and max gain values reported in Appendix S8)

Site	Model	AUC	Sensitivity at HSI threshold	Specificity at HSI threshold
Tripod Fire (Washington)	Tripod logistic regression	0.795	0.964	0.344
	Toolbox logistic regression	0.795	0.893	0.636
	Star Gulch logistic regression	0.750	0.893	0.630
	2-variable Mahalanobis *D*^2^	0.825	0.929	0.623
	3-variable Mahalanobis *D*^2^	0.782	0.964	0.669
	4-variable Mahalanobis *D*^2^	0.825	0.821	0.766
	3-variable Maxent	0.828	0.821	0.686
	ΔNBR-only Maxent	0.795	0.857	0.639
Silver-Toolbox fires (Oregon)	Tripod logistic regression	0.696	0.909	0.726
	Toolbox logistic regression	0.696	0.727	0.697
	Star Gulch logistic regression	0.693	0.727	0.686
	2-variable Mahalanobis *D*^2^	0.653	0.841	0.702
	3-variable Mahalanobis *D*^2^	0.665	0.864	0.687
	4-variable Mahalanobis *D*^2^	0.674	0.659	0.664
	3-variable Maxent	0.721	0.705	0.757
	ΔNBR-only Maxent	0.696	0.727	0.703
Star Gulch Fire (Idaho)	Tripod logistic regression	0.730	0.917	0.749
	Toolbox logistic regression	0.730	0.778	0.691
	Star Gulch logistic regression	0.762	0.778	0.683
	2-variable Mahalanobis *D*^2^	0.646	0.889	0.708
	3-variable Mahalanobis *D*^2^	0.737	0.917	0.688
	4-variable Mahalanobis *D*^2^	0.727	0.556	0.689
	3-variable Maxent	0.741	0.722	0.728
	ΔNBR-only Maxent	0.730	0.778	0.696

**Figure 4:**
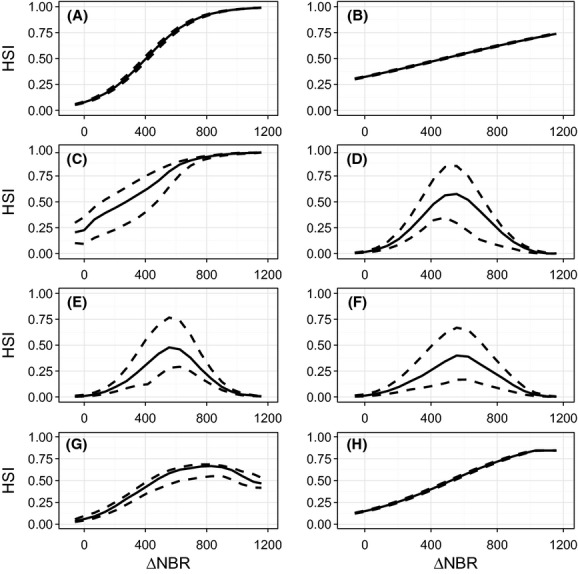
Dose–response plots depicting habitat suitability index (HSI) relationships with delta normalized burn ratio (ΔNBR; Tripod Fire weighted logistic regression [WLR; A], Star Gulch Silver-Toolbox fires WLR [B], Fire WLR [C], 2-variable Mahalanobis [D], 3-variable Mahalanobis [E], 4-variable Mahalanobis [F], 3-variable Maxent [G], ΔNBR-only Maxent [H]). Solid lines depict median HSI values; broken lines depict 25th and 75th median-unbiased percentiles.

All models described relationships with burn severity, but distance models (Mahalanobis *D*^2^) described peaked relationships with ΔNBR, whereas resource selection models described relatively monotonic and positive relationships (Fig. 4). Four models described relationships with prefire canopy cover variables (Appendix S8), assigning lower HSIs to pixels with minimal prefire canopy cover (Appendix S9). Of these four models, however, the WLR model described a strong positive relationship, whereas the two distance models and the 3-variable Maxent model described weaker plateauing relationships. Three models lacking prefire canopy cover variables (Tripod Fire WLR, 2-variable Mahalanobis *D*^2^, and ΔNBR-only Maxent models) nevertheless also described somewhat positive albeit weak relationships with prefire canopy cover. Three models (2- and 4-variable Mahalanobis *D*^2^ and 3-variable Maxent models) described relatively weak relationships with cosine aspect that varied among models (Appendix S9).

### Ensemble predictions and model agreement

**Figure 5:**
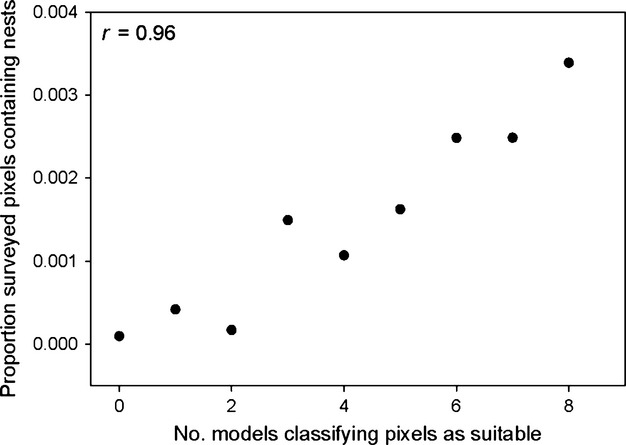
Relationship between ensemble predictions of suitability (no. models classifying surveyed pixels [*n* = 76,655] as highly suitable) and the proportion of these pixels in which nests were located. Sample sizes for ensemble prediction levels (0–8) ranged from 3622 to 20,578 pixels. *r* = Pearson's correlation coefficient.

**Figure 6:**
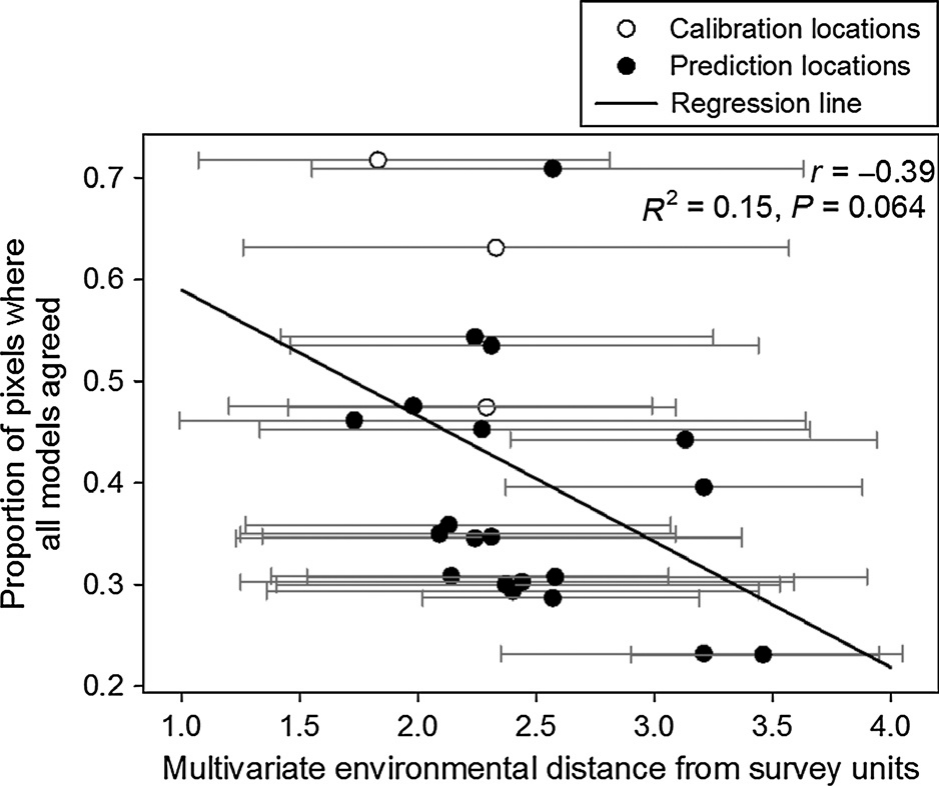
Relationship between habitat suitability model agreement and median environmental distance from surveyed units. Median distance and 95% median-unbiased intervals were calculated from surveyed units to 23 wildfire locations (outside survey unit boundaries at the three calibration locations [Tripod, Silver-Toolbox, and Star Gulch locations] and 20 eastside Montana locations). Model agreement was measured as the proportion of pixels within each burned location consistently classified either as highly suitable or as low suitability habitat by all 8 models.

The proportion of pixels predicted highly suitable varied among models and locations (Table 5). Despite differences among models, we found complete agreement among all 8 models at 52.8% of pixels outside survey units at Tripod, Silver-Toolbox, and Star Gulch locations, including 40.0% of pixels at eastside Montana fires. Within survey units, the number of models predicting pixels as highly suitable related strongly and linearly with the proportion of pixels in which nests were located (*R*^2^ = 0.92, *P* < 0.0001; Fig. 5). Environments outside survey units at Tripod, Silver-Toolbox, and Star Gulch locations were similar to environments within survey units, whereas some eastside Montana locations were quite different (Appendix S4). Model agreement declined with environmental distance from surveyed units (Fig. 6). Environmental distance scores indicated the sampled environmental range (median *D* and 95th percentiles = 1.90 [0.97–3.01]; *n* = 15,000 pixels sampled from within surveyed units) was narrower than the entire range across which models were applied (2.35 [1.13–3.81]; *n* = 128,687 pixels from all locations). Models mainly agreed on their identification of low suitability habitat. Only 19.7% of the area outside survey units was classified highly suitable by all 8 models, whereas 27.7% of this area was consistently classified low suitability.

**Table 5:** Proportion of wildfire locations predicted highly suitable by habitat suitability models for Black-backed Woodpecker (*Picoides arcticus*). For model descriptions, see Methods and Appendix S8. WLRs, weighted logistic regression models; Mahal, Mahalanobis models, and Mxnt, Maxent models. Ensemble predictions described the proportion of each location classified highly suitable by at least 1 model (most liberal) and 8 models (most conservative). Values for the model calibration locations (Tripod, Silver-Toolbox, and Star Gulch) were compiled outside survey unit boundaries

Wildfire location	Tripod WLR	Silver-Toolbox WLR	Star Gulch WLR	2-var Mahal	3-var Mahal	4-var Mahal	3-var Mxnt	ΔNBR-only Mxnt	1 model	8 models
Tripod	0.331	0.337	0.564	0.217	0.349	0.305	0.283	0.328	0.687	0.161
Silver-Toolbox	0.292	0.303	0.253	0.333	0.278	0.295	0.234	0.286	0.489	0.157
Star Gulch	0.292	0.299	0.224	0.302	0.260	0.280	0.259	0.287	0.418	0.180
Ahorn	0.691	0.698	0.776	0.300	0.467	0.489	0.557	0.688	0.855	0.338
Big Creek	0.392	0.405	0.243	0.350	0.238	0.258	0.231	0.386	0.558	0.146
Cascade	0.630	0.638	0.837	0.265	0.630	0.528	0.483	0.625	0.891	0.357
Cigarette Rock	0.705	0.711	0.660	0.291	0.395	0.423	0.505	0.701	0.796	0.308
Davis	0.670	0.673	0.810	0.271	0.584	0.601	0.576	0.668	0.857	0.445
Derby	0.451	0.461	0.321	0.404	0.263	0.292	0.316	0.445	0.589	0.185
Diamond Complex	0.671	0.685	0.167	0.625	0.020	0.030	0.371	0.664	0.775	0.017
Fool Creek	0.732	0.739	0.769	0.305	0.434	0.400	0.544	0.728	0.871	0.292
Hicks Park	0.422	0.431	0.493	0.359	0.415	0.505	0.334	0.419	0.719	0.215
Jungle	0.805	0.810	0.815	0.364	0.431	0.478	0.612	0.802	0.899	0.360
Madison Arm	0.711	0.725	0.002	0.724	0.000	0.000	0.342	0.705	0.769	0.000
Meriwether	0.647	0.657	0.749	0.416	0.648	0.636	0.547	0.642	0.839	0.468
Middlefork	0.770	0.778	1.000	0.337	0.594	0.394	0.643	0.766	1.000	0.360
Mill	0.164	0.173	0.078	0.231	0.119	0.137	0.117	0.159	0.330	0.052
Nanny Creek	0.671	0.683	0.752	0.366	0.540	0.505	0.488	0.666	0.869	0.349
Passage Falls	0.506	0.516	0.466	0.293	0.283	0.287	0.266	0.500	0.654	0.160
Powder River	0.398	0.416	0.091	0.444	0.000	0.005	0.214	0.391	0.557	0.001
Skyland	0.625	0.633	0.881	0.323	0.478	0.459	0.574	0.622	0.923	0.329
Watt Draw	0.499	0.517	0.030	0.456	0.010	0.012	0.146	0.490	0.604	0.006
Wicked Creek	0.730	0.737	0.581	0.367	0.257	0.282	0.403	0.726	0.807	0.204

## Discussion

Ensemble predictions at eastside Montana locations acknowledged uncertainties in our knowledge of Black-backed Woodpecker ecological relationships. Ideally, data from locations of management interest would be used to develop or validate particular models for use at those locations. In situations requiring habitat conservation for disturbance specialists, however, such data may be unattainable within the necessary time frame needed to guide management decisions. This challenge parallels that of conservation planning in the face of future climate changes (Araújo and New 2007). By accounting for alternative hypotheses regarding habitat suitability relationships for nesting and foraging Black-backed Woodpeckers during the breeding season, ensemble predictions presented here may represent the best available information for guiding management of recently burned forests. In areas where models agree, habitat suitability can be seen as relatively certain because predictions of suitability (or unsuitability) in these areas are robust to inaccuracies associated with particular hypotheses represented by different models. Conversely, model disagreement suggests where future data collection efforts might be focused to test competing hypotheses and thereby further ecological knowledge of this species.

Agreement among all 8 models occurred at a substantial proportion of Montana locations. Limitations in currently available data may limit the predictive performance of any model (e.g., consider prediction of relationship C in Fig. 3). Additional data sampling a broader range of environments could improve our general understanding of ecological relationships and thus our ability to identify suitable nesting habitat with greater certainty. Alternatively, local adaptation may cause variation in habitat relationships across this species' range (e.g., Bonnot et al. 2009; Morrison 2012), in which case models tailored to specific subpopulations or ecotypes may be required for accurate prediction. Regardless, deviations from observed habitat selection patterns will more likely occur in environments that differ from sampled locations (Aarts et al. 2013). Consistent with declining confidence in model predictions, model agreement declined when applied in environments that diverged from sampled environments. Furthermore, the number of models classifying habitat as highly suitable was linearly related to the proportion of pixels containing nests, paralleling a desirable property of individuals models in the context of prediction (Jiménez-Valverde et al. 2013).

Knowledge to be gained becomes apparent when considering alternative ecological processes that potentially underlie relationships hypothesized by different models. The models selected for prediction described observed data reasonably well and were consistent with known nesting habitat requisites of Black-backed Woodpecker (Saab et al. 2009, 2011). Fire is clearly an important requisite across most of this species' range, so all models described relationships with burn severity. Resource selection models generally described positive relationships, whereas distance models described optimal burn severities. Black-backed Woodpeckers favor relatively severe fires. An upper limit to severity beyond which fire could be detrimental (e.g., when too many snags are entirely consumed) has yet to be determined for fire specialists and for the promotion of ecological resiliency. Future conditions driven by climate change, however, could reveal severity thresholds for fire-associated species because a warming climate is expected to increase fire severity (Whitlock et al. 2003). Our models did not consistently indicate a burn severity level within the sampled environmental range beyond which habitat suitability declines. Nevertheless, an optimal severity could become more evident with additional data from eastside Montana, where wildfires burned more severely than at sampled locations. Densities of postfire snags and prefire trees also likely determined habitat suitability for nesting Black-backed Woodpeckers. Previous work documents preferences for relatively high snag densities (Russell et al. 2007; Saab et al. 2009), and persistence of snags, relating to persistence of suitable nest sites, also increases with snag density (Russell et al. 2006). Whether habitat suitability continues to increase or plateaus from moderate to high tree densities remains unclear. A greater prefire tree density may also elevate burn severity (Lentile et al. 2006; but see Turner et al. 1999), for which an optimum may exist as discussed above. The north–south orientation of the slope (represented by cosine aspect) was not a predominant component of our models. Modeled relationships with this feature could reflect real ecological relationships with variation in moisture and thus vegetation structure, although these relationships may be sufficiently accounted for by metrics of snag density. We nevertheless retained this element in our ensemble of models for further evaluation as additional data are collected.

Assuming all selected models represent realistic descriptions of suitable habitat, model disagreement can in part reflect differences between alternative ecological hypotheses, providing direction for future research. Some ensemble prediction studies and tools have emphasized either resource selection models (Marmion et al. 2009; Thuiller et al. 2009) or distance models and related techniques (Aragón et al. 2010). Because these two classes of models represent divergent paradigms, their predictions will tend to diverge outside sampled environments. Variation in model complexity can also cause model disagreement. Parsimonious models may avoid being over-fitted to particular datasets, potentially favoring general applicability (Wenger and Olden 2012). Nevertheless, complex models could reflect more of a species' ecology and therefore should not be discounted until empirically evaluated. Inclusion of models varying in underlying structure and complexity in one's ensemble will naturally lead to reduced agreement as predictions are applied in environments that differ from sampled locations. Assuming models represent viable alternative hypotheses, this tendency of ensemble predictions is consistent with our declining confidence in predictions in un-sampled environments and is therefore desirable. Although our ensemble predictions represented a range of hypotheses, they by no means included the full range of potential predictions inferred from our data. Developing a set of models that represents the full range of possibilities may be impractical. We therefore suggest starting with models that capture a variety of ecological hypotheses and then adjusting one's ensemble as field testing confirms, refutes, or suggests new hypotheses.

### Limitations and additional considerations

Various limitations are worth considering when applying these models. Although AUCs suggested all models were at least somewhat informative for discriminating high from low suitability habitats, other indicators suggested relatively poor performance by certain models. The Silver-Toolbox WLR model improved AIC_c_ by only 2.1 over an intercept-only model, suggesting poor explanatory power. Median nest HSIs from selected Mahalanobis *D*^2^ models in this study were lower than those reported elsewhere (Rotenberry et al. 2006; Preston et al. 2008; Knick et al. 2013), which may be undesirable for predictive power (Rotenberry et al. 2006). Additionally, model selection results for WLRs at all locations indicated uncertainty in the relative importance of particular habitat features. Surveyed units at the Silver-Toolbox fires were burned more severely and included a somewhat narrower severity range compared with areas outside these units and to other sampled locations (see Appendix S4). Stronger evidence for a burn severity relationship at other locations, where a wider range of severities were sampled, corroborated the importance of this habitat feature. WLR model selection results and lower percent variable contributions to Maxent models, however, indicated uncertainty in the importance of relationships with the other three habitat variables.

Variables used here served as proxies for other features (prefire canopy cover for tree density and cosine aspect for climate). Variables that more directly measure features of interest could improve explanatory and predictive power of the models and may be more definitively related to habitat suitability. A completely continuous metric of prefire canopy cover (i.e., percent canopy cover rather than proportion of neighborhood over 40%) might be a better proxy for postfire snag density, but such data were not available at all locations. In addition, inclusion of variables describing additional factors not currently represented, such as tree size or species composition, could improve model performance. The growing sources of remotely sensed data (e.g., Lefsky et al. 2002; Recio et al. 2013) will likely provide additional environmental data useful for modeling Black-backed Woodpecker habitat suitability. Nevertheless, for a generally applicable model or ensemble of models, the need for remotely sensed data that are consistently available throughout this species' range may inevitably limit model performance.

Our models describe fine-scale variation in nesting habitat suitability, which describes where we expect Black-backed Woodpeckers to initiate nests and subsequently forage assuming they are present at a wildfire location. Other factors besides habitat suitability, such as dispersal limitation (Hoyt and Hannon 2002), are also important to species occurrence patterns. To locate unpredictably available burned-forest habitat, Black-backed Woodpeckers must be adept dispersers, which is evident in the lack of genetic structure across wide regions (Pierson et al. 2010). Some dispersal limitation is evident, however, particularly for females (Pierson et al. 2010). We sampled at least two distinct populations; Black-backed Woodpeckers in Oregon belong to a genetically distinct population that likely extends into California, whereas Idaho and western Montana birds belong to a separate population (Pierson et al. 2010). Thus, an understanding of coarser scale distributional patterns may require consideration of variability in population dynamics among regions. Differentiation among populations may also cause local adaptation (Pearman et al. 2008; Bonnot et al. 2009), possibly further complicating application of models for predicting habitat selection patterns outside sampled regions (Morrison 2012). We found some consistency in apparent habitat relationships across at least two distinct populations (burn severity parameters for the three WLRs were similar in direction and magnitude). Nonetheless, collection of location-specific data for validating ensemble predictions would be particularly desirable in un-sampled regions (e.g., eastside Montana).

As is commonly the case, our models assume habitat relationships are spatially and temporally static and are not influenced by biotic interactions (i.e., competition and predation). Such assumptions can limit model performance (Zurell et al. 2009). Black-backed woodpeckers are attracted to burned forests because of increases in nesting and foraging opportunities provided by snags (Nappi et al. 2009; Saab et al. 2007, 2009). Reduced competition due to increased opportunity for nesting likely explains, in part, why certain cavity-nesting birds are attracted to burns (Russell et al. 2003). Additionally, nest predator populations may temporarily decline following a burn, potentially relaxing predation pressure (Russell et al. 2010). Variation among regions in nest predation pressure or snag availability in unburned forests may modulate the attractiveness of burns for cavity-nesting species and thus the black-backed woodpecker's relationship with burn severity. In addition to local adaptation, dispersal limitation, variability in demography, and biotic interactions, relatively simple mechanisms can induce variability in habitat relationships (Aarts et al. 2013). Finally, because nesting habitat for black-backed woodpecker is ephemeral (Saab et al. 2007), the predictive value of static habitat suitability models is necessarily limited. With additional data, models could be refined to allow some variability in habitat relationships either via interactions among predictors (Aarts et al. 2013), hierarchical structuring (Pearson and Dawson 2003), or inclusion of temporal dynamics (Mieszkowska et al. 2013). Until such refinements are implemented, however, the relatively simple models presented here provide a useful first approximation for focusing efforts to conserve habitat for black-backed woodpeckers (see also arguments by Pearson and Dawson 2003).

### How predictions can help guide habitat management

**Figure 7:**
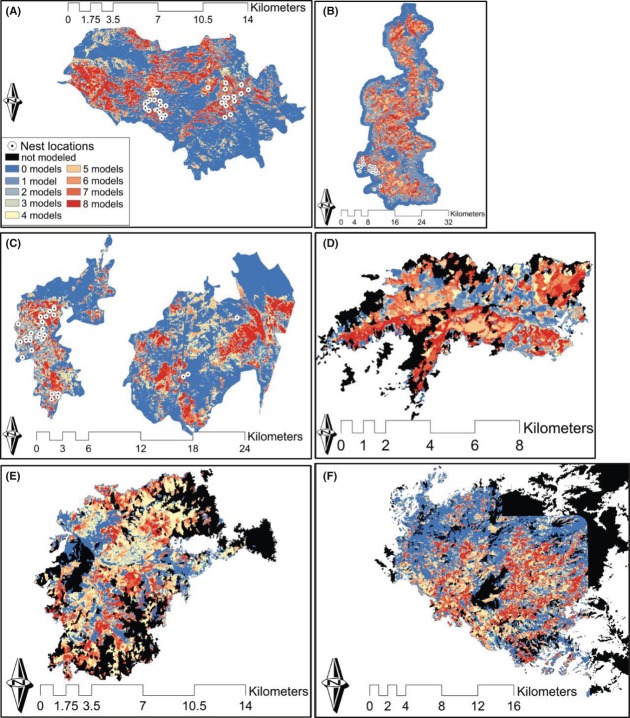
Ensemble habitat suitability predictions for nesting Black-backed Woodpeckers at Star Gulch (A), Tripod (B), Silver-Toolbox (C), and four eastside Montana wildfire locations (Cascade [D], Wicked Creek and Passage Falls [fires are adjacent; E], and Derby [F]). Blue pixels were classified highly suitable by a minority and red pixels by a majority of models. Black areas at eastside Montana locations were not evaluated because those areas were either not dry, mixed-conifer forest or lacked the necessary data for making predations.

Ensemble predictions generated here reflect available knowledge of Black-backed Woodpecker nesting habitat relationships in burned forests. If postfire management objectives include wildlife habitat conservation, managers could conservatively restrict salvage logging in areas identified highly suitable by liberal ensemble predictions, which would include most wildfire locations in eastside Montana. For a more balanced approach, land managers could conserve areas predicted highly suitable by more models (e.g., red-shaded areas; Fig. 7). Ideally, managers would follow an adaptive management strategy, whereby they would initially manage habitat conservatively (e.g., restrict salvage logging in areas predicted highly suitable by ≥3 models), while conducting surveys to corroborate predictions and refine models as necessary. Our ensemble predictions are best applied to dry mixed-conifer forests, but data collected in other forest types could be used to test model applicability across a broader range of forests. Additionally, remotely sensed variables derived from prefire conditions could be used to model nesting habitat in unburned areas with an increased risk of burning. Such work could inform development of conservation strategies that account for changing fire regimes expected as a result of climate change (Whitlock et al. 2003).

## Supporting Information

**Appendix S1.** List of eastside Montana wildfires where models were applied to predict habitat suitability for nesting Black-backed Woodpeckers.

**Appendix S2.** Correlations (Pearson's *r*) between environmental variables used to develop habitat suitability models for black-backed woodpeckers.

**Appendix S3.** List of steps taken for Mahalanobis *D*^2^ model development and selection.

**Appendix S4.** Descriptive statistics (mean, SD) for environmental variables for wildfire locations.

**Appendix S5.** AIC_c_ values for top weighted logistic regression models.

**Appendix S6.** Parameter estimates (maximum likelihood estimator ± SE) for selected weighted logistic regression models describing nest habitat relationships for Black-backed Woodpeckers.

**Appendix S7.** Evaluation scores calculated using validation data for candidate partitioned Mahalanobis *D*^2^ models.

**Appendix S8.** Model descriptions and HSI thresholds selected for classifying map pixels as either highly suitable or low suitability habitat for Black-backed Woodpecker nests.

**Appendix S9.** Dose–response plots (derived from zonal statistics) depicting HSI (habitat suitability index) relationships with environmental variables used to model habitat suitability for nesting black-backed woodpeckers.
