# Supplementary material for: Ensemble modeling to predict habitat suitability for a large-scale disturbance specialist
Source: Ecol Evol. 2013 Oct 6;3(13):4348–64. doi: 10.1002/ece3.790 (PMC3856736; doi:10.1002/ece3.790)

**APPENDICES**

Appendix S1. Eastside Montana wildfires where models were applied to predict habitat suitability for nesting Black-backed Woodpeckers.

| National Forest | Fire Name | Ignition Year | Approximate Extent (ha) | |
| --- | --- | --- | --- | --- |
| Custer | Watt Draw | 2006 | 9,623 |  |
|  | Powder River Complex | 2007 | 4,633 |  |
|  | Cascade | 2008 | 4,152 |  |
|  | Diamond Complex | 2011 | 18,234 |  |
|  | Mill | 2011 | 2,490 |  |
|  |  |  |  |  |
| Gallatin | Big Creek | 2006 | 4,533 |  |
|  | Derby | 2006 | 42,596 |  |
|  | Jungle | 2006 | 7,738 |  |
|  | Passage Falls | 2006 | 2,964 |  |
|  | Hicks Park (WH Complex) | 2007 | 752 |  |
|  | Madison Arm | 2007 | 1,489 |  |
|  | Wicked Creek (WH Complex) | 2007 | 8,751 |  |
|  |  |  |  |  |
| Helena | Meriwether | 2007 | 7,225 |  |
|  | Davis | 2010 | 860 |  |
|  |  |  |  |  |
| Lewis & Clark | Cigarette Rock | 2006 | 845 |  |
|  | Nanny Creek | 2006 | 968 |  |
|  | Ahorn | 2007 | 19,261 |  |
|  | Fool Creek | 2007 | 21,527 |  |
|  | Middle Fork | 2007 | 460 |  |
|  | Skyland | 2007 | 14,945 |  |

Appendix S2. Correlations (Pearson’s *r*) between environmental variables used to develop habitat suitability models for black-backed woodpeckers and with multivariate environmental distances (Mahalanobis *D*). Correlations were calculated for 128,687 30×30-m pixels representing a maximum of 5,000 pixels selected randomly from each of 3 calibration locations and 23 locations targeted for prediction (outside survey unit boundaries at calibration locations, and at 20 eastside Montana wildfire locations). See Table 2 for full variable names and descriptions.

|  | COSASP | ΔNBR | LocCC | LandCC |
| --- | --- | --- | --- | --- |
| ΔNBR | 0.154 | 1.000 |  |  |
| LocCC | 0.152 | 0.315 | 1.000 |  |
| LandCC | -0.093 | 0.249 | 0.630 | 1.000 |
| Environmental distance | 0.236 | 0.325 | -0.240 | -0.396 |

Appendix S3. Steps taken for developing and selecting partitioned Mahalanobis *D*^2^ models using nest site data from 3 locations: Tripod (*n* = 28), Silver-Toolbox (*n* = 44), Star Gulch (*n* = 36).

**Step 1. Re-sampling**: Draw without replacement 28 nest pixels from each location (n = 84 total) 100 times.

**Step 2. Further divide re-sampled datasets for cross-validation:** Assign as equally as possible the 84 nest pixels in each re-sampled dataset into one of five data divisions.

**Step 3. Identify 500 calibration datasets and their associated validation datasets.** A calibration dataset is any combination of 4 data divisions, the remaining data division being the validation dataset.

**Step 4. Variance partitioning.** To each calibration dataset, apply principal components analysis to generate variance partitions for each of four variable combinations. This generated 10 partitions for 10 models.

**Step 5. Calculate HSIs for model replicates.** For each model and each calibration dataset, calculate the *D^2^* statistic and re-scale using a Χ^2^ distribution to generate HSIs for validation data.

**Step 6. Generate performance scores.** For each validation dataset, calculate median HSIs and AUCs. Average these across the 500 validation datasets for each model.

**Step 7. Model selection.** Select the model that optimizes median HSI and AUC scores for validation data.

**Step 8. Calculation of final HSIs:** Calculate HSIs for selected model without cross-validation for use in evaluation and selection of classification thresholds.

Appendix S4. Environmental descriptive statistics (mean, s.d.) for wildfire locations. Values for within versus outside survey units at Star Gulch (Idaho), Tripod (Washington), and Silver-Toolbox (Oregon) are presented separately. Environmental distances (Mahalanobis *D*) from survey plots (median [25^th^ percentile, 75^th^ percentile]) are also presented. Values were averaged across a max of 5,000 30×30-m pixels randomly drawn from each location.

| Wildfire site | COSASP | ΔNBR | LocCC | LandCC | Environmental distance |
| --- | --- | --- | --- | --- | --- |
| Tripod w/in survey units | 0.07, 0.69 | 296.47, 246.58 | 0.78, 0.36 | 0.73, 0.17 | 2.04 (1.24, 2.83) |
| Silver-Toolbox w/in survey units | -0.06, 0.69 | 370.45, 201.13 | 0.61, 0.43 | 0.56, 0.16 | 1.80 (1.06, 2.85) |
| Star Gulch w/in survey units | -0.13, 0.69 | 306.64, 248.57 | 0.48, 0.42 | 0.47, 0.14 | 1.85 (1.07, 2.81) |
| Tripod outside survey units | -0.05, 0.69 | 300.57, 286.97 | 0.77, 0.36 | 0.77, 0.19 | 2.29 (1.45, 3.09) |
| Silver-Toolbox outside survey units | 0.25, 0.63 | 249.15, 197.56 | 0.34, 0.41 | 0.34, 0.22 | 2.33 (1.26, 3.57) |
| Star Gulch outside survey units | -0.11, 0.69 | 308.95, 246.54 | 0.48, 0.42 | 0.47, 0.14 | 1.83 (1.07, 2.81) |
| Ahorn | 0.01, 0.67 | 569.46, 303.87 | 0.69, 0.42 | 0.66, 0.20 | 2.24 (1.23, 3.37) |
| Big Creek | -0.18, 0.65 | 336.31, 201.16 | 0.37, 0.42 | 0.32, 0.14 | 2.24 (1.42, 3.25) |
| Cascade | -0.12, 0.81 | 478.42, 250.25 | 0.76, 0.36 | 0.71, 0.15 | 2.14 (1.38, 3.06) |
| Cigarette Rock | -0.05, 0.66 | 570.54, 306.68 | 0.54, 0.45 | 0.49, 0.17 | 2.31 (1.34, 3.37) |
| Davis | 0.20, 0.73 | 536.94, 294.43 | 0.81, 0.34 | 0.71, 0.10 | 2.13 (1.27, 3.07) |
| Derby | 0.12, 0.69 | 357.39, 245.11 | 0.36, 0.41 | 0.33, 0.12 | 2.31 (1.46, 3.44) |
| Diamond Complex | 0.28, 0.65 | 451.24, 192.54 | 0.27, 0.36 | 0.12, 0.08 | 3.21 (2.35, 4.05) |
| Fool Creek | -0.14, 0.66 | 571.37, 279.92 | 0.65, 0.43 | 0.60, 0.23 | 2.40 (1.36, 3.44) |
| Hicks Park | 0.01, 0.55 | 390.54, 272.14 | 0.62, 0.43 | 0.49, 0.13 | 1.73 (0.99, 3.64) |
| Jungle | 0.20, 0.64 | 648.01, 279.19 | 0.63, 0.44 | 0.56, 0.19 | 2.44 (1.25, 3.59) |
| Madison Arm | 0.73, 0.39 | 428.00, 133.31 | 0.08, 0.23 | 0.08, 0.05 | 3.46 (2.90, 3.95) |
| Meriwether | 0.10, 0.74 | 486.89, 251.48 | 0.72, 0.40 | 0.63, 0.15 | 1.98 (1.20, 2.99) |
| Middlefork | -0.29, 0.65 | 535.82, 234.05 | 0.96, 0.16 | 0.94, 0.02 | 2.57 (2.02, 3.19) |
| Mill | 0.21, 0.67 | 238.79, 138.67 | 0.33, 0.41 | 0.24, 0.16 | 2.57 (1.55, 3.63) |
| Nanny Creek | -0.26, 0.57 | 503.36, 256.01 | 0.66, 0.42 | 0.61, 0.21 | 2.09 (1.25, 3.09) |
| Passage Falls | -0.01, 0.75 | 412.92, 273.21 | 0.41, 0.44 | 0.41, 0.15 | 2.27 (1.33, 3.66) |
| Powder River | 0.28, 0.67 | 362.91, 177.28 | 0.21, 0.35 | 0.11, 0.06 | 3.13 (2.39, 3.94) |
| Skyland | 0.22, 0.7 | 534.94, 317.10 | 0.88, 0.27 | 0.76, 0.19 | 2.37 (1.40, 3.53) |
| Watt Draw | 0.12, 0.71 | 348.02, 170.42 | 0.14, 0.30 | 0.09, 0.08 | 3.21 (2.37, 3.88) |
| Wicked Creek | 0.08, 0.68 | 554.56, 289.07 | 0.39, 0.42 | 0.38, 0.14 | 2.58 (1.53, 3.9) |

Appendix S5. Model selection results for top weighted logistic regression models. Models described Black-backed Woodpecker nest habitat preferences at three wildfire locations: Tripod (WA), Silver-Toolbox (OR), and Star Gulch (ID). Models within 2 AIC_c_ units of the best model and intercept-only models are presented. See Table 2 for variable abbreviations.

| Location | Model | AICc | ΔAICc |
| --- | --- | --- | --- |
| Tripod | ΔNBR^S^ | 146.0 | 0.0 |
|  | ΔNBR + LandCC | 146.6 | 0.6 |
|  | COSASP + ΔNBR | 147.4 | 1.4 |
|  | Intercept only | 204.5 | 58.5 |
|  |  |  |  |
| Silver-Toolbox | ΔNBR^S^ | 260.6 | 0.0 |
|  | ΔNBR + LandCC | 260.8 | 0.2 |
|  | COSASP + ΔNBR | 262.3 | 1.6 |
|  | LandCC | 262.5 | 1.8 |
|  | Intercept only | 262.7 | 2.1 |
|  |  |  |  |
| Star Gulch | ΔNBR + LandCC^S^ | 108.9 | 0.0 |
|  | ΔNBR | 109.9 | 1.0 |
|  | COSASP + ΔNBR + LandCC | 110.3 | 1.4 |
|  | ΔNBR + LocCC | 110.5 | 1.6 |
|  | Intercept only | 117.2 | 8.3 |

^S^Models selected for ensemble predictions.

Appendix S6. Parameters (maximum likelihood estimator ± s.e.) for selected weighted logistic regression models describing nest habitat relationships for Black-backed Woodpecker at three wildfires: Tripod (Washington), Toolbox-Silver fires (Oregon), and Star Gulch (Idaho). “n/a” indicates parameters that were excluded.

| Model | Intercept | ΔNBR | LandCC |
| --- | --- | --- | --- |
| Tripod | -2.49 ± 0.45 | 0.0062 ± 0.0010 | n/a |
| Silver-Toolbox | -0.74 ± 0.38 | 0.0015 ± 0.0007 | n/a |
| Star Gulch | -3.13 ± 1.04 | 0.0037 ± 0.0012 | 3.15 ± 1.73 |

Appendix S7. Evaluation scores calculated using validation data for candidate partitioned Mahalanobis *D*^2^ models. Models are sorted from highest to lowest AUC (area under the receiver-operating curve). *k* = the partition number, which equals the number of principal components included in the model.

| Variables | *k* | median nest HSI | AUC |
| --- | --- | --- | --- |
| COSASP, ΔNBR, LocCC, LandCC | 4^A,B^ | 0.445 | 0.716 |
| ΔNBR, LocCC, LandCC | 3^A,B^ | 0.449 | 0.708 |
| COSASP, ΔNBR | 2^A,B^ | 0.439 | 0.704 |
| ΔNBR | 1^A^ | 0.427 | 0.672 |
| COSASP, ΔNBR, LocCC, LandCC | 3 | 0.432 | 0.616 |
| ΔNBR, LocCC, LandCC | 2 | 0.435 | 0.604 |
| ΔNBR, LocCC, LandCC | 1 | 0.435 | 0.596 |
| COSASP, ΔNBR, LocCC, LandCC | 1 | 0.423 | 0.590 |
| COSASP, ΔNBR, LocCC, LandCC | 2 | 0.406 | 0.588 |
| COSASP, ΔNBR | 1 | 0.475^C^ | 0.577 |

^A^*k*_max_ models equivalent to unpartitioned *D*^2^ models

^B^selected model for ensemble predictions

^C^maximum median nest HSI

Appendix S8. Model descriptions and HSI thresholds selected for classifying map pixels as either highly suitable or low suitability habitat for Black-backed Woodpecker nests. Thresholds were selected so as to maximize predictive gain (sensitivity – [1 − specificity]) across all sites where nest location data were collected. See Table 2 in manuscript for variable names and descriptions.

| Model | Model descriptions | HSI threshold that maximized predictive gain | Predictive gain at HSI threshold |
| --- | --- | --- | --- |
| Tripod Fire weighted logistic regression | WLR fitted to Tripod data w/ ΔNBR | 0.43 | 0.41 |
| Silver-Toolbox fires weighted logistic regression | WLR fitted to Silver-Toolbox data w/ ΔNBR | 0.45 | 0.40 |
| Star Gulch Fire weighted logistic regression | WLR fitted to Star Gulch data w/ ΔNBR and LandCC | 0.43 | 0.37 |
| 2-variable Mahalanobis *D*^2^ | Multivariate distance model based on COSASP and ΔNBR | 0.32 | 0.33 |
| 3-variable Mahalanobis *D*^2^ | Multivariate distance model based on ΔNBR, LocCC, and LandCC | 0.17 | 0.39 |
| 4-variable Mahalanobis *D*^2^ | Multivariate distance model based on COSASP, ΔNBR, LocCC, and LandCC | 0.17 | 0.42 |
| 3-variable Maxent | Maxent model allowing linear, quadratic, and product relationships with ΔNBR, COSASP, and LocCC | 0.41 | 0.42 |
| ΔNBR-only Maxent | Maxent model allowing only a linear relationship with ΔNBR | 0.37 | 0.40 |

Appendix S9. Dose-response plots depicting black-backed woodpecker HSI (habitat suitability index) relationships with environmental variables. Solid lines = median; dotted lines = 25^th^ and 75^th^ median unbiased percentiles. Plots reflect realized HSI relationships that do not control for inter-correlations between habitat variables. dNBR = ΔNBR. HSI relationships with all environmental variables are shown; asterisks indicate variables included in each model.

Appendix S8A. Tripod Fire weighted logistic regression model HSI relationships.


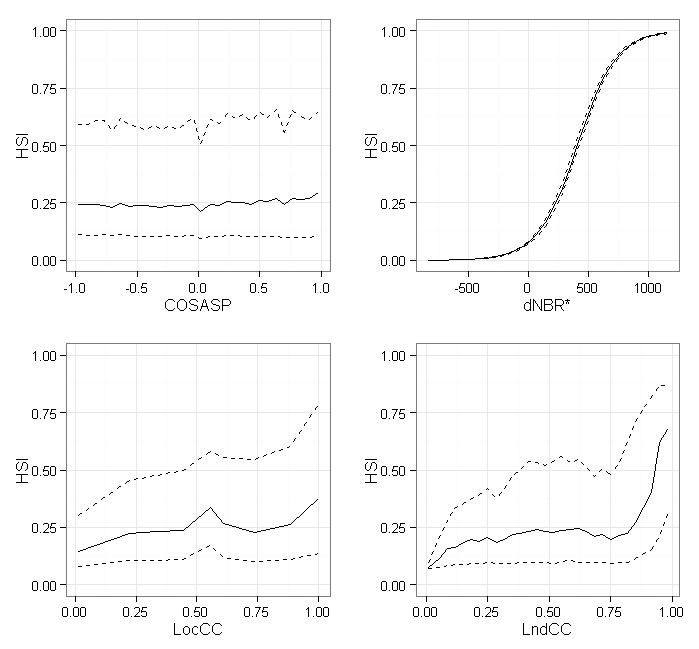


Appendix S9B. Silver-Toolbox fires weighted logistic regression model HSI relationships.


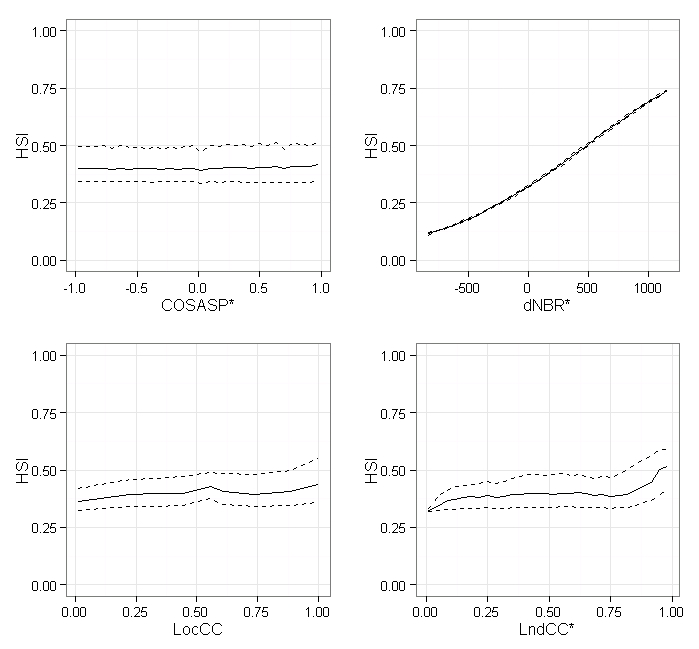


Appendix S9C. Star Gulch Fire weighted logistic regression model HSI relationships.


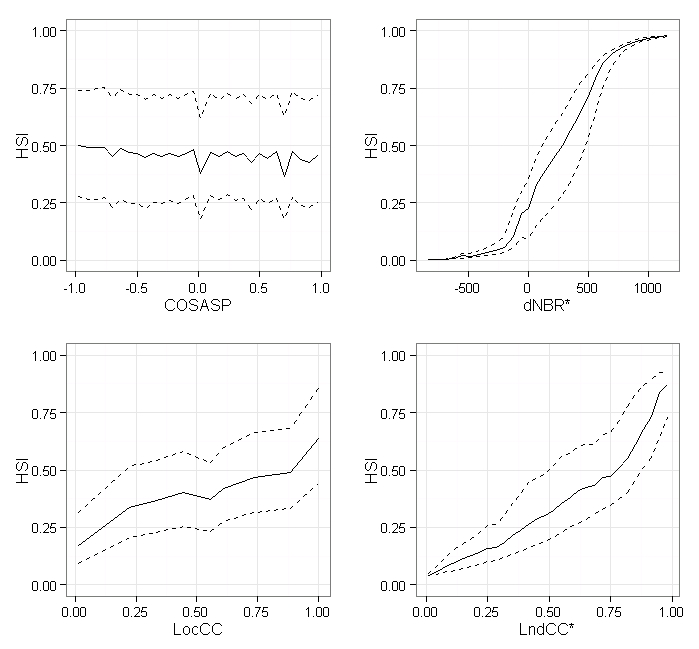


Appendix S9D. 2-variable Mahalanobis *D*^2^ model HSI relationships.


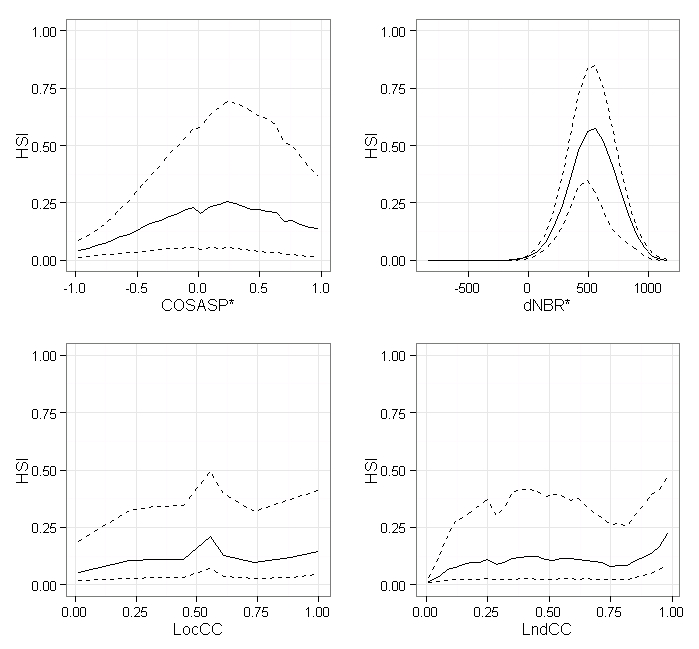


Appendix S9E. 3-variable Mahalanobis *D*^2^ model HSI relationships.


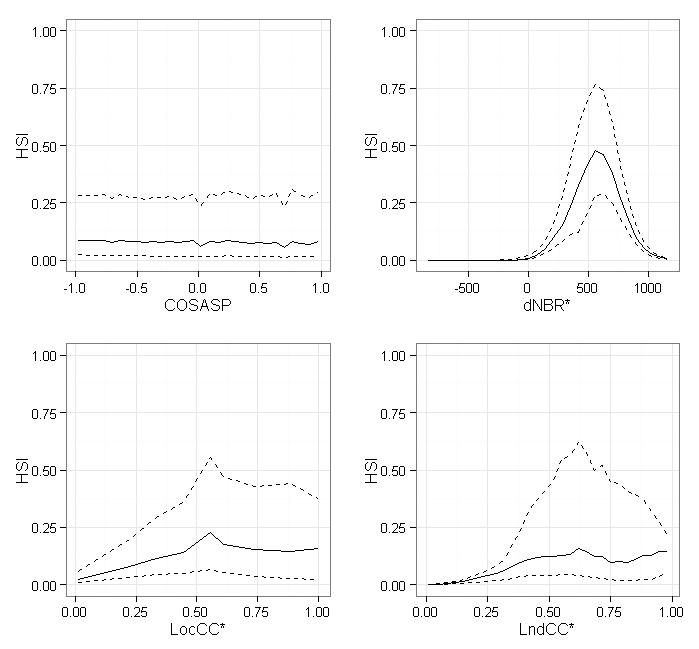


Appendix S9F. 4-variable Mahalanobis *D*^2^ model HSI relationships.


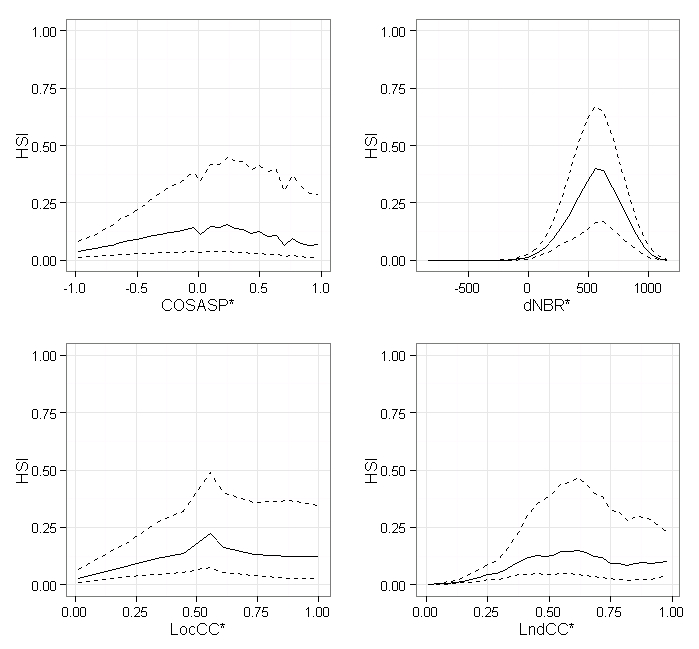


Appendix S9G. 3-variable Maxent model HSI relationships.


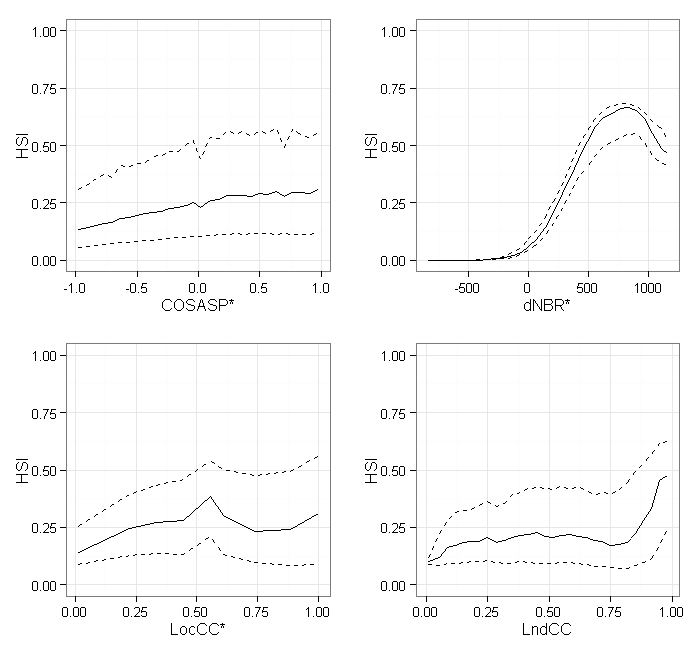


Appendix S9H. ΔNBR-only Maxent model HSI relationships.


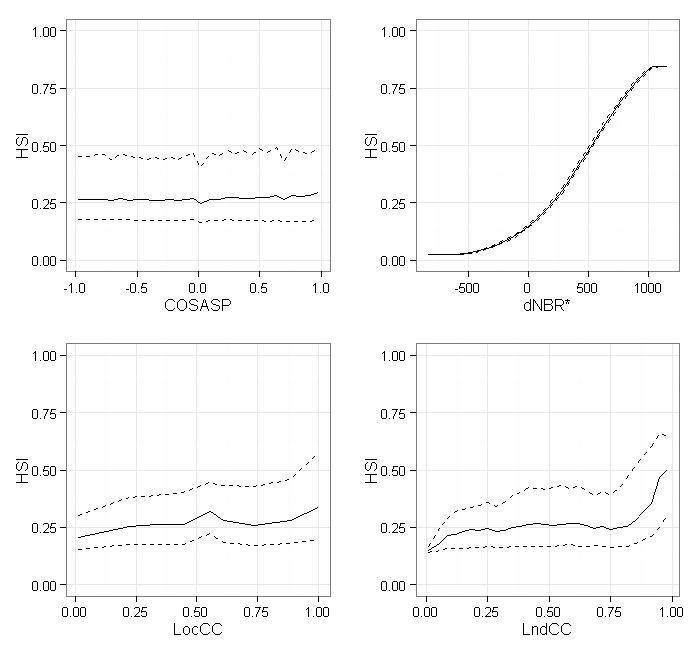

Supplement: Supplementary file 1 [file ece30003-4348-SD1.docx]
